# Navigating Cardiac Myxomas: A Compelling Case of a Giant Atrial Myxoma With Embolic Complications

**DOI:** 10.7759/cureus.81801

**Published:** 2025-04-06

**Authors:** Varshika Ramakrishnan Chandra Babu, Sharwani Hirurkar, Sarah Al-Fayyadh, Omprakash Reddy Desireddygari, Prakash Nanjaiah

**Affiliations:** 1 Department of Cardiothoracic Surgery, University Hospitals of North Midlands National Health Service (NHS) Trust, Stoke-on-Trent, GBR

**Keywords:** atypical presentation, benign cardiac tumors, cardiac myxoma, massive atrial myxoma, pulmonary embolism

## Abstract

Cardiac myxomas are the most common primary cardiac tumors, predominantly affecting the left atrium and often seen in women aged 30-60. They can present diagnostic and therapeutic challenges, especially in atypical demographics. If left untreated, myxomas can lead to severe morbidity due to embolic events and intracardiac obstruction. Herein, we describe a rare presentation of an exceptionally large left atrial myxoma, emphasizing the clinical, diagnostic, and surgical considerations required to manage such complex cases effectively. A 61-year-old man with a history of poorly controlled hypertension and hyperlipidemia presented with dyspnea, lower extremity edema, and syncope, as an acute manifestation and was found to have a large, mobile mass measuring 9.5 × 4.5 cm in the left atrium, intermittently prolapsing into the mitral valve orifice during diastole on transthoracic echocardiography, with a provisional diagnosis of myxoma. Additionally, a chest computed tomography scan revealed a subsegmental pulmonary embolus, with the left-sided myxoma being the most plausible cause. Given the tumor size and embolic risk, the mass was managed surgically via a median sternotomy approach. Histopathology confirmed a benign cardiac myxoma, characterized by stellate and myxoid cells within a myxomatous stroma. Postoperatively, the patient's symptoms resolved, and a 12-week follow-up echocardiography showed no residual mass. This case underscores the critical importance of considering cardiac myxoma in the differential diagnosis of acute cardiac symptoms, particularly when embolic phenomena are concurrent. The case highlights the necessity for prompt surgical intervention to mitigate the risk of embolic complications and to restore normal hemodynamic function. Despite the benign nature of myxomas, their potential for severe complications necessitates a high index of suspicion and timely intervention.

## Introduction

Cardiac myxomas are rare, oval or round-shaped, mobile, pedunculated or sessile intracardiac neoplasms. Despite their rarity, they have an incidence of up to one per million individuals [1]. Some studies show that these benign intracardiac tumors occur more frequently in women than in men, reporting a ratio of 3:1 between the two sexes [2]. It is estimated that more than 75% of myxomas originate in the left atrium either at the mitral annulus or the fossa ovalis border of the interatrial septum, while 20% arise from the right atrium, and the remaining 5% stem from both atria and the ventricle [3]. The peak incidence of atrial myxomas occurs between the fourth and sixth decades of life [4]. A French study conducted by Pinede et al. showed that myxomas can lead to a triad of complications, the first being obstruction (67%), followed by emboli (29%), with or without constitutional symptoms (34%) [5], which include fever, malaise, anorexia, arthralgia, and weight loss [3]. Morphologically, cardiac myxomas are categorized into polypoid and papillary forms. Polypoid myxomas are often associated with obstructive symptoms, whereas papillary myxomas are more commonly linked to embolic and neurological complications [5]. The diagnostic modality of choice is echocardiography, particularly transesophageal echocardiography (TOE), which provides superior visualization of tumor size, site, attachment, and mobility, and also grossly differentiates the myxoma from vegetation or a thrombus [6,7]. Other diagnostic tests include coronary angiography and magnetic resonance imaging. The treatment of choice is surgical excision, which is aimed at complete resection and has excellent outcomes [8].

We present a rare case of a giant atrial myxoma in a 61-year-old man, presenting with advanced New York Heart Association (NYHA) class IV symptoms. Unlike typical myxomas, this tumor caused severe mitral valve obstruction, pulmonary embolism, and systemic complications, including pulmonary hypertension, bilateral pleural effusions, and ascites. Furthermore, the incidental finding of pancreatic cysts added diagnostic complexity. The rarity of such a large tumor with multifaceted complications and unique presentation makes this case particularly compelling for study and discussion.

## Case presentation

**Figure 1:**
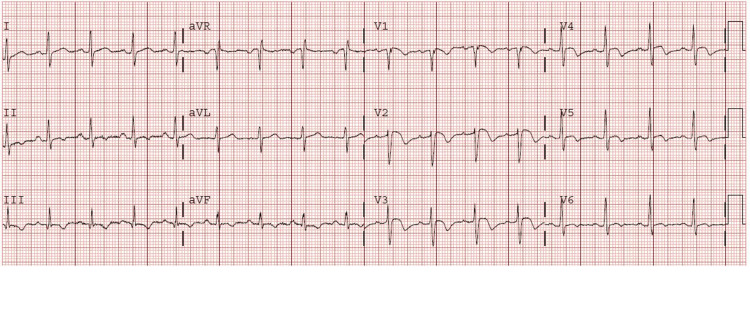
An electrocardiogram revealing T-wave inversions in nearly all chest leads (V1-V6) and S1Q3T3 changes aVR: augmented vector right; aVL: augmented unipolar lead; aVF: augmented vector foot

**Figure 2:**
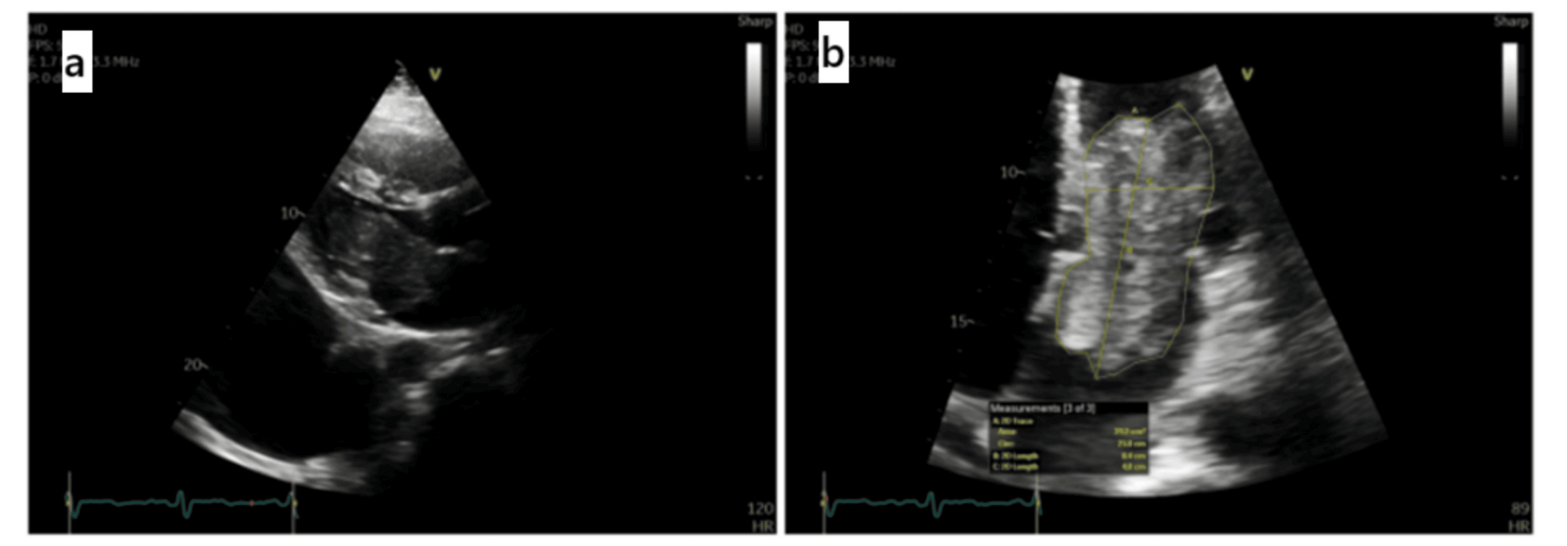
(a) Large pleural effusion. (b) Left atrial mass protruding into left ventricle

A 61-year-old man presented to the Emergency Department with persistent central chest pain for approximately four hours associated with supraventricular tachycardia (SVT), shortness of breath on exertion, lower extremity edema, and a history of syncope. The patient’s episode of SVT led to significant hemodynamic instability by increasing heart rate, which compromised left ventricular filling. This further exacerbated the restriction of mitral inflow caused by the myxoma's ball-valve effect, ultimately approaching complete cardiac output failure and necessitating urgent stabilization. On physical examination, his heart sounds were normal, but he displayed shortness of breath on exertion and bilateral lower leg swelling. His past medical history was significant for poorly controlled hypertension and hyperlipidemia. Upon clinical examination, the patient’s blood pressure was recorded at 150/70 mmHg, and his pulse rate was 86 beats per minute, while other vital signs were stable. Initial electrocardiogram showed T-wave inversion in nearly all chest leads (V1-V6) and S1Q3T3 changes, indicative of right heart strain (Figure 1). Concurrently elevated troponin I levels (55 ng/L) raised suspicion for non-ST-elevated myocardial infarction. As his symptoms persisted, he underwent an emergent transthoracic echocardiography (TTE), which revealed an irregularly shaped large mobile mass in the left atrium, measuring 9.5 × 4.5 cm, occluding the mitral valve and protruding into the left ventricle (Figure 2b). Other parameters obtained from the echocardiogram include bilateral pleural effusions (measuring up to 9 cm) (Figure 2a), ascites, and left ventricular ejection fraction of 50% with evidence of severe pulmonary hypertension. The development of bilateral pleural effusions and ascites was most likely secondary to right heart failure, which arose as a consequence of the mitral valve obstruction caused by the atrial myxoma. These findings raised concern for a cardiac myxoma, later confirmed by a two-dimensional TOE. TOE provided additional diagnostic value over TTE, enabling a better characterization of the myxoma's mobility, highlighting the ball-valve effect, which contributed to the observed mitral valve obstruction. No other valve abnormalities or lesions were detected, and the only significant finding related to the heart valves was mild mitral regurgitation. Given the presence of ascites, a computed tomography (CT) scan of the abdomen and pelvis was done, which revealed an incidental 16-mm cystic lesion in the pancreas. Chest CT identified subsegmental pulmonary emboli; however, the source of the embolism could not be definitively attributed to the left-sided myxoma. The left-sided myxoma remains the most plausible source.

Following a multidisciplinary team discussion, urgent surgical intervention was planned to reduce the risk of embolization from the tumor. While the patient was awaiting surgery, he developed an episode of acute chest pain, warranting an urgent CT coronary angiogram, which showed a moderate disease in the mid-left anterior descending artery, but negative fractional flow reserve, indicating no significant ischemia.

**Figure 3:**
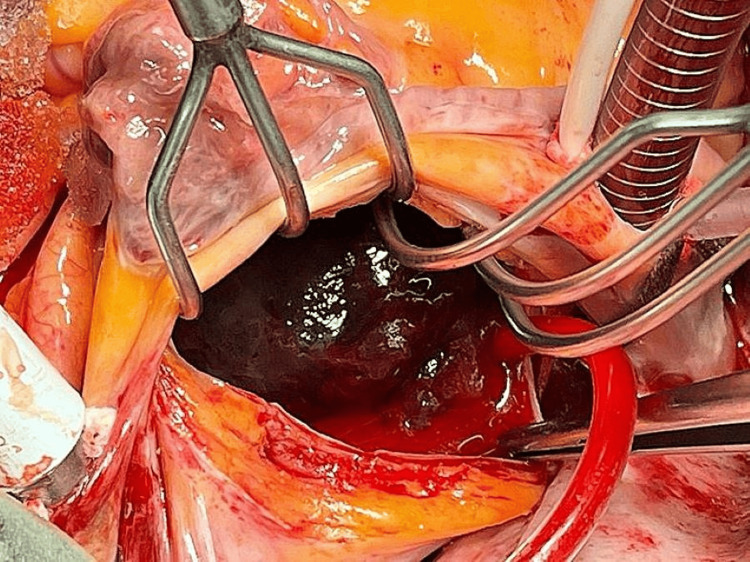
Intraoperative image of the sessile mass attached to the left (lateral) atrial wall with a broad base, located approximately 1 cm from the right fibrous trigone of the mitral valve

**Figure 4:**
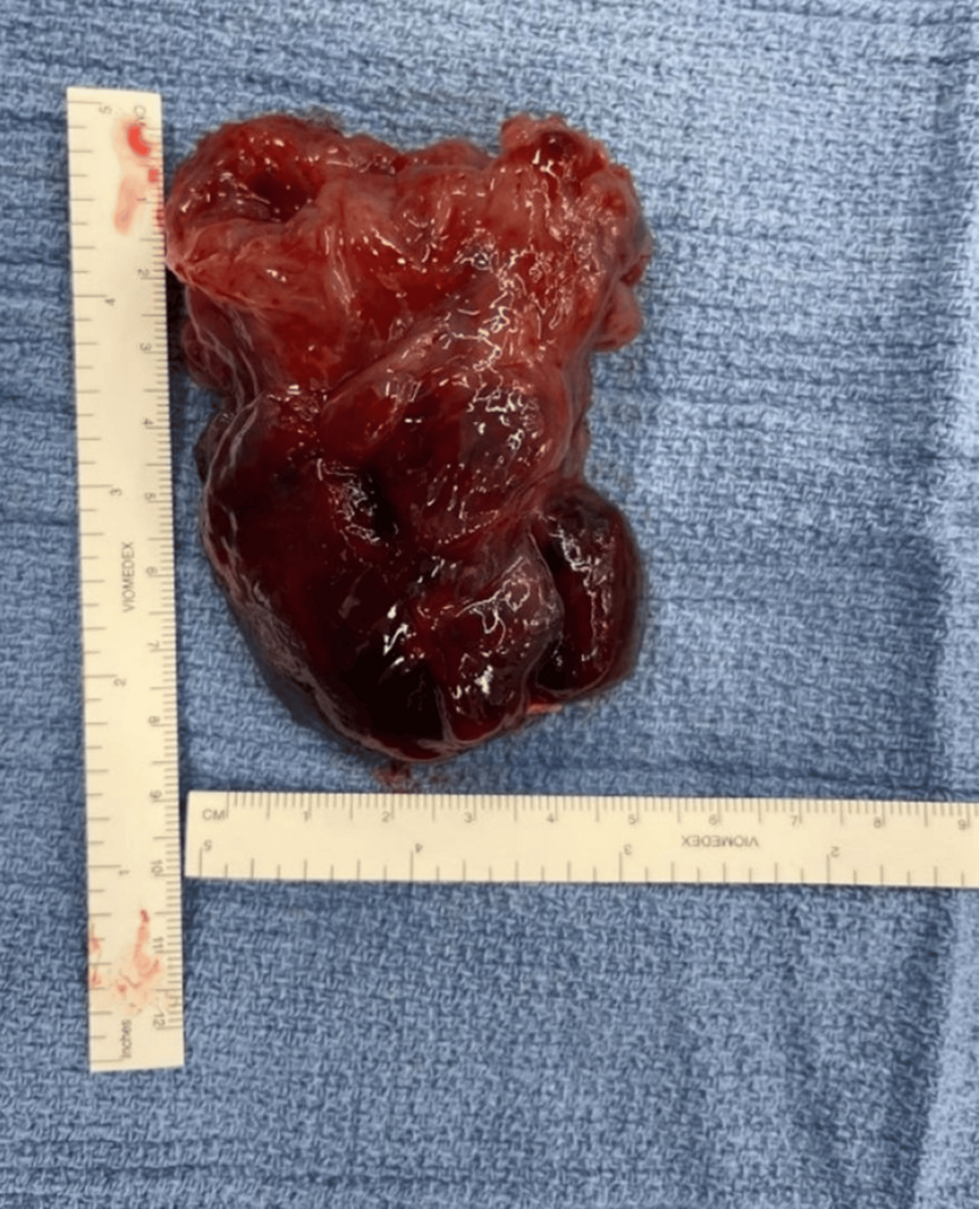
Operative finding of a fully excised left atrial mass measuring approximately 8.5 × 4.5 cm

The surgical approach was performed by median sternotomy, aorto-bicaval cardiopulmonary bypass, and antegrade cold-blood cardioplegia. After the left atrium was accessed via Sondergaard’s groove, a large, lobulated, round mass with a gelatinous consistency was visualized within the left atrium. The mass was sessile, with a broad base on the interatrial septum, located approximately 1 cm from the right fibrous trigone of the mitral valve, and the left atrium was mildly dilated (Figure 3). The atrial mass was excised en bloc with its base attached to the interatrial septum; the resulting septal defect was closed directly using a 3/0 pledgeted Prolene suture, without the need for patch repair, and was sent for histopathological examination (Figure 4). While the precise dimensions of the excised septal area were not formally recorded, the direct closure was feasible due to the size and location of the defect. During surgery, both pleural spaces were accessed, and approximately 2 L of straw-yellow fluid was drained from the pleural cavities. The pleural effusions were likely to be transudative in nature, secondary to right heart failure resulting from the obstructive effects of the myxoma. Post-bypass TOE showed the adequacy of mass excision and no significant mitral or tricuspid valve regurgitation or any atrial septal defects. While the preoperative echocardiography and CT scan indicated the mass to be approximately 9.5 x 4.5 cm, the intraoperative findings showed a mass measuring 8.5 x 4.5 cm. This minor discrepancy may be attributed to variations in measurement techniques.

**Figure 5:**
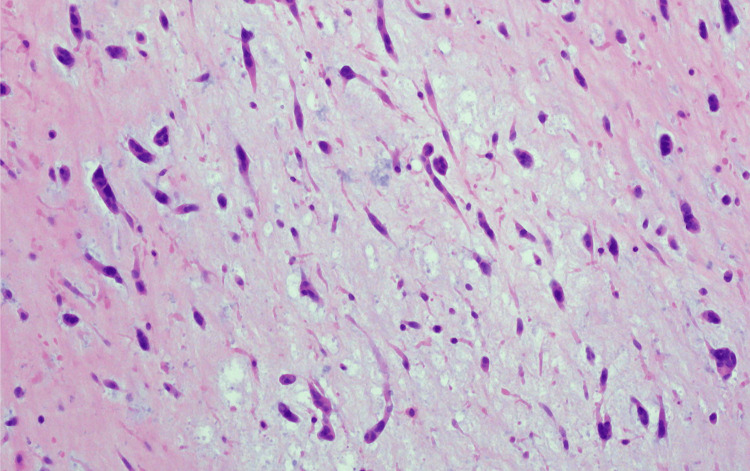
Histopathological image of benign atrial myxoma, obtained from local laboratory: dispersed stellate and spindle cells in a fibromyxoid stroma

The postoperative course was uneventful, and the patient was discharged six days after surgery. Initially, the patient was started on lifelong warfarin therapy due to the subsegmental pulmonary embolism, but this was later discontinued following a stable three-month follow-up. Histopathological examination was consistent with a benign atrial myxoma, characterized by dispersed stellate to plump spindled cells with abundant cytoplasm set within a fibromyxoid stroma (Figure 5). Myxomas are typically classified into two main categories: polypoid and papillary, based on their morphological appearance. Although the present case does not definitively categorize the myxoma as one type over the other, the overall presentation, including the ball-valve effect and associated complications such as mitral valve obstruction, may provide clues as to the type of myxoma. Polypoid myxomas, typically more mobile and larger, tend to present with greater embolic risk. At a 12-week follow-up, the patient reported substantial resolution of his symptoms and marked improvement in functional class from NYHA class IV preoperatively to class II. An echocardiogram was performed, which confirmed the absence of any residual mass or structural abnormalities. The findings suggested no recurrence of the myxoma or any other significant cardiac issues, contributing to the patient's overall improvement. Postoperative surveillance is essential for monitoring for recurrence of the myxoma, and follow-up transthoracic echocardiograms (TTEs) are typically performed at 3, 6, and 12 months after surgery. Annual echocardiographic evaluations are recommended thereafter to detect any recurrence, given the known risk of myxoma recurrence and the potential for associated complications. These follow-up intervals help to ensure early identification of any structural changes or recurrence.

## Discussion

Cardiac myxomas, though rare, are the most common primary tumors of the heart. The size of atrial myxomas differs widely among patients but generally ranges from 2 to 6 cm [9]. One of the largest reported left atrial myxomas measured 9.6 × 8.2 × 6.8 cm and caused pulmonary hypertension and mitral valve obstruction [10]. The left atrial myxoma in our case measures 8.5 x 4.5 cm. Although slightly smaller in width compared to the largest documented case [10], the tumor’s length and clinical impact were notable, which included mitral valve obstruction, severe pulmonary hypertension, and systemic embolization. The hemodynamic instability in this patient was multifactorial. The large left atrial myxoma functioned as a ball-valve, intermittently occluding the mitral valve inlet, severely restricting left ventricular filling. This was further exacerbated by the onset of SVT, which shortened diastolic filling time, reducing preload and nearly leading to complete cardiac output failure. This combination of mechanical obstruction and tachycardia-induced inadequate ventricular filling explains the patient’s severe presentation, necessitating urgent intervention.

Larger myxomas are associated with an increased risk of obstruction, embolization, and hemodynamic compromise. In our case, the tumor’s size and location led to significant mitral valve obstruction, severe pulmonary hypertension, and systemic embolization, further emphasizing the importance of early detection. Approximately 30%-40% of atrial myxomas present with systemic embolization, most commonly affecting the brain, limbs, or coronary arteries. In our case, the patient had segmental pulmonary emboli, which, although uncommon in left-sided myxomas, can occur. TTE is the first-line modality for detecting cardiac myxomas, with a sensitivity of 95%. However, TOE offers superior visualization, particularly for tumors near the atrial septum or those with small attachments [6,7]. In this case, TOE confirmed the presence of a large, mobile left atrial mass with characteristics consistent with a myxoma. Given the high risk of embolization and mitral valve obstruction, early surgical resection is the gold standard for atrial myxomas. Larger left or right atrial myxomas and all ventricular myxomas are generally better approached using median sternotomy [11]. Complete excision with a margin on the interatrial septum is recommended to prevent recurrence, which occurs in 2%-3% of cases [12]. Our patient underwent successful en bloc resection via median sternotomy, with no recurrence observed on follow-up echocardiography. This case highlights the importance of considering cardiac myxomas in patients presenting with unexplained dyspnea, systemic embolization, or signs of mitral valve obstruction. Prompt echocardiographic evaluation and early surgical intervention are crucial to preventing life-threatening complications. To assess for myxoma recurrence, routine postoperative echocardiographic evaluation is necessary [13]. Furthermore, the broader clinical implications of cardiac myxomas include the risks of systemic embolization, cardiac valve dysfunction, and recurrence, highlighting the need for comprehensive preoperative, postoperative, and long-term surveillance to optimize patient outcomes.

## Conclusions

In conclusion, it is vital to note that ruling out atypical causes of common presentations like dyspnea, systemic embolization, and mitral valve obstruction is vital for accurate diagnosis and early intervention. The profound nature of the patient's presentation was primarily due to the combination of hemodynamic compromise and embolization. The hemodynamic instability was a direct result of the obstruction caused by the large myxoma, leading to impaired left ventricular filling and a risk of cardiac output failure. Additionally, the embolization events, including the subsegmental pulmonary embolism, further worsened the patient's clinical condition and prompted urgent intervention. The combination of these two factors, mechanical obstruction and embolic complications, made the case particularly severe and required immediate surgical management to prevent life-threatening outcomes.

The timely use of multimodal imaging played a crucial role in identifying the tumor, reinforcing the need for early diagnosis and intervention. Given the risk of further embolization, early surgical intervention remains the most effective strategy to mitigate fatal complications. Ultimately, this case serves as a reminder that clinicians should maintain a high index of suspicion for cardiac tumors in patients with unexplained cardiopulmonary symptoms. Beyond emphasizing the need for early detection, this case also highlights the challenges in differentiating myxomas from other cardiac masses.

## References

[1] Islam AK (2022). Cardiac myxomas: a narrative review. World J Cardiol.

[2] Sido V, Volkwein A, Hartrumpf M (2023). Gender-related outcomes after surgical resection and level of satisfaction in patients with left atrial tumors. J Clin Med.

[3] Nguyen T, Vaidya Y (2023). Atrial Myxoma. https://www.ncbi.nlm.nih.gov/books/NBK556040/.

[4] Yu K, Liu Y, Wang H, Hu S, Long C (2007). Epidemiological and pathological characteristics of cardiac tumors: a clinical study of 242 cases. Interact Cardiovasc Thorac Surg.

[5] Pinede L, Duhaut P, Loire R (2001). Clinical presentation of left atrial cardiac myxoma. A series of 112 consecutive cases. Medicine (Baltimore).

[6] Swartz MF, Lutz CJ, Chandan VS, Landas S, Fink GW (2006). Atrial myxomas: pathologic types, tumor location, and presenting symptoms. J Card Surg.

[7] Iyer P, Aung MM, Awan MU, Kososky C, Barn K (2016). A case of large atrial myxoma presenting as an acute stroke. J Community Hosp Intern Med Perspect.

[8] Thyagarajan B, Kumar MP, Patel S, Agrawal A (2017). Extracardiac manifestations of atrial myxomas. J Saudi Heart Assoc.

[9] Reynen K (1995). Cardiac myxomas. N Engl J Med.

[10] Buyukates M, Aktunc E (2008). Giant left atrial myxoma causing mitral valve obstruction and pulmonary hypertension. Can J Surg.

[11] MacGillivray TE, Reardon MJ (2020). Surgical treatment of benign cardiac tumors. Oper Tech Thorac Cardiovasc Surg.

[12] Jeswani D, Kannan P, Pachaiyappan P (2022). Repeated recurrence of non-familial cardiac myxoma. J Am Coll Cardiol.

[13] Qin W, Wang L, Chen X, Liu P, Wang R (2014). Left ventricular myxoma: a case report. J Biomed Res.

